# Sex Differences in the Impact of Physical Activity Across the Spectrum of Cardiovascular Disease

**DOI:** 10.1007/s11886-026-02361-9

**Published:** 2026-03-18

**Authors:** Yamini Levitzky, Karina Gonzalez Carta, Lavanya Kondapalli, Elijah Davis, Sharon Andrade-Bucknor

**Affiliations:** 1 Division of Cardiology, Mass General Brigham at Newton-Wellesley Hospital, Newton, MA USA; 2 Division of Cardiovascular Diseases, Mayo Clinic Rochester, Rochester, MN USA; 3 Division of Cardiology, University of Colorado Anschutz Medical Campus, Denver, CO USA; 4 College of Arts and Sciences, Washington University in St. Louis, St. Louis, MO USA; 5 Division of Cardiology, University of Miami, 1321 NW 14th Street, Suite 510, Miami, FL 33125 USA

**Keywords:** Exercise response, Cardiac rehabilitation, Sex differences, Women, Physical activity

## Abstract

**Purpose of Review:**

To synthesize current evidence on sex-related differences in physiological responses, clinical outcomes, and implementation of exercise training across different cardiovascular disease phenotypes.

**Recent Findings:**

Physical activity provides benefits regardless of the patient’s sex, improves cardiovascular health and overall quality of life. Studies have found that women experience a relatively greater mortality risk reduction compared to men.

**Summary:**

Women gain greater cardiovascular and mortality benefits fromphysical activity than men, even with less exercise. Biological and hormonaldifferences shape distinct exercise responses and outcomes between sexes.Cardiac rehabilitation is highly effective for women but faces low referral andparticipation rates. Tailored, sex-specific exercise recommendations areneeded to optimize cardiovascular health in women.

## Introduction

Cardiovascular disease (CVD) remains a leading cause of morbidity and mortality worldwide, with chronic coronary disease (CCD) affecting over 20 million Americans. While men generally exhibit a higher prevalence of CCD, the true burden in women is likely underestimated due to differences in symptom presentation and diagnostic challenges. Sex-specific factors, including hormonal changes, pregnancy-related complications, and female-predominant conditions, play a crucial role in shaping the clinical landscape of CVD and underscore the need for nuanced approaches to management.

Physical activity is a cornerstone of cardiovascular health, endorsed by major professional societies for both prevention and management of CCD. However, men and women respond differently to exercise, influenced by physiological, molecular, and behavioral factors. These differences extend to exercise preferences, intensity, and outcomes, with women often participating in lower-intensity activities and experiencing unique cardiovascular benefits and risks.

Understanding the sex differences on the impact of physical activity across the spectrum of cardiovascular disease is essential for optimizing care, improving outcomes, and tailoring recommendations. This document explores the molecular, physiological, and clinical dimensions of these differences, highlighting the importance of individualized exercise prescriptions and comprehensive risk factor modification.

## Sex-Specific Benefits of Exercise

The benefits of exercise on all-cause and cardiovascular mortality have been well established in multiple large-scale studies [1]. The differential benefit of volume of exercise in women vs. men is less widely known. In a recent prospective study of physical activity in > 400,000 US adults, comparing regular physical activity in women vs. men, there was a 24% (HR: 0.76; 95% CI: 0.73–0.80) vs. 15% (HR: 0.85; 95% CI: 0.82–0.89) decrease in all-cause mortality, and 36% vs. 14% risk reduction in cardiovascular mortality. In addition to aerobic activity, muscle strengthening physical activity 3x per week was also associated with a 2-fold greater relative reduction in all-cause mortality and a 3-fold relative reduction in cardiovascular mortality in women [2]. Furthermore, with strength training, females show greater relative improvements which is predictive of mortality more so than muscle mass [3]. Women reached their maximum survival benefit from moderate-vigorous physical activity at 140 min per week compared with men, who require 300 min per week to derive this same benefit [2]. Similarly, analyses utilizing the National Health and Nutrition Examination Survey (NHANES) public database spanning from 2007 -2008 to 2017–2018 with 29,172 participants, female sex was again associated with maximal risk reduction in mortality at lower non occupational or leisure time physical activity than required in men [4]. These findings are particularly relevant in the context of the gender disparity in physical activity levels observed between women and men, which has been attributed to multiple factors such as limited time, lack of motivation, and various environmental, social, and cultural influences [5].

## Biological Differences of Sex-Specific Exercise Response

### Molecular and Physiologic Effects of Exercise on Vasculature

It is well-established that women experience cardiovascular events at a later age than men, which has in large part been ascribed to the protective effect of estrogen in premenopausal women [6]. While a detailed discussion of the molecular pathways is beyond the scope of this paper, estrogen plays a central role in maintaining endothelial health and vascular function. Estrogen enhances endothelial nitric oxide synthase activity, facilitates vasodilation, and provides antioxidant protection which ultimately improves vascular function as well as lowering vascular resistance [6, 7]. As estrogen levels decline after menopause, there is a decrease in endothelial repair and alteration of the inflammatory, atherogenic, and thrombotic milieu [8]. Flow-mediated dilation (FMD) of the brachial artery, a widely used tool to assess endothelial function, correlates with coronary function and predicts incident CVD events [7, 9, 10]. Across adulthood, women show greater FMD compared with men through their 60s, but FMD in postmenopausal women drops to nearly half of that seen in pre-menopausal women [7]. An umbrella review examining the impact of exercise training on FMD in general adult populations (nearly 40% women) noted that there is evidence of benefit of both aerobic and resistance training on FMD [11]. Similarly, a systematic review focused specifically on post-menopausal women found net benefit in macrovascular function assessed by FMD as well as biomarker measures of endothelial function [12].

These baseline sex differences in vascular physiology also influence how women and men respond to exercise. Biomarkers that are responsive to exercise, such as natriuretic peptides show sex-specific kinetics and baseline levels. For example, women have higher resting NT-proBNP concentrations than men regardless of menopausal status, which is thought to be due to lower levels of circulating androgens [13]. In a small study of healthy, untrained volunteers, a single session of exercise was shown to increase NT-proBNP in both men and women, but only men demonstrated a significant association between echo measures of diastolic dysfunction and rise in NT-pro BNP [14]. Autonomic regulation also differentiates women from men; estrogen has anti-sympathetic effects and vasodilatory effects which reduces cardiac work and contributes to lower resting blood pressure, blunted vasoconstrictor response, and a higher frequency of post-exercise hypotension [15]. Women rely more on oxygen extraction from peripheral tissues rather than increased cardiac output, whereas men rely more heavily on stroke volume and systemic oxygen delivery due to hemoglobin and increased muscle mass [16]. These sex-specific distinctions in hemodynamic responses may in part explain why women experience different aerobic capacity gains, post-exercise recovery, and cardiovascular protection compared with men.

### Structural and Functional Differences

Many structural and physiological differences exist between females and males which contribute to the differential effect of exercise in healthy individuals as well as in cardiovascular disease states.

Women have smaller muscle size and a higher proportion of type II fibers, whereas men more commonly exhibit type I fibers. These differences contribute to sex-specific muscle energy metabolism, with men demonstrating greater glycolytic capacity and women greater whole-muscle oxidative capacity. Lower VO2 max is seen in women which is largely due to smaller heart size with smaller stroke volumes, stroke volume index and lower hemoglobin mass [17, 18]. Greater vascular conductance and blood flow during exercise with higher capillary density has also been noted in women; however, these findings have not been entirely consistent across studies, in part due to the relatively small number of women in most cardiovascular studies [4, 17].

Women demonstrate a higher vasodilator response and in elderly women, there is increased arterial stiffness, increased arterial elastance index and hypertensive response. Pulmonary factors such as smaller lung size and lower diffusion capacity also play a role in women [19].

Hormonal changes influence the effects of exercise leading to decreased muscle strength and balance in postmenopausal women compared with premenopausal women of the same age. However, hormone therapy has not been shown to be protective against impaired physical performance [17].

## Coronary Artery Disease

Exercise is a key component of maintaining cardiovascular health and preserving quality of life in patients with CCD in combination with guideline-directed medical therapy, treatment of hypertension, lipids, nutrition counseling, and psychosocial support. As such, cardiac rehabilitation-guided exercise programs are endorsed by both the American Heart Association and the American College of Cardiology for management of patients with CCD [20]. However, as men and women respond differently physiologically to exercise, identifying an exercise program sufficient in intensity and duration for a demonstrable cardiovascular benefit is not a one-size-fits-all endeavor. Pre- vs. post-menopausal status undoubtedly plays a role. Choice of exercise may also be relevant - women tend to participate in activities such as yoga, aerobics, and dance, while men are more likely to participate in higher intensity activities, such as hiking, racket sports, basketball and football [21]. 

Chronic coronary disease is a heterogeneous group of diagnoses, ranging from obstructive and non-obstructive disease, with or without prior myocardial infarction and/or revascularization, or chronic anginal syndromes [20]. While the prevalence is generally higher in men than in women, particularly over the age of 60, it is likely that the prevalence in women is underestimated due to the variable presentations of ischemic symptoms in women [20]. Even accounting for this underestimation, the prevalence of CCD in non-Hispanic black women (7.2%) approaches that of non-Hispanic white men (8.7%). Recognition that hormonal differences combined with non-traditional risk factors that primarily affect women such as adverse pregnancy outcomes (including gestational diabetes, pregnancy-induced hypertension and use of assisted reproduction), early menopause, or female-predominant conditions (such as autoimmune diseases or breast cancer treatment) are now thought to play a role in sex differences in CVD presentation and call for nuanced and sex-specific recommendations for optimal management [8].

### Plaque Characteristics

Differences in atherosclerotic plaque burden are observed in men and women with CAD. Men develop atherosclerosis at a younger age with more vulnerable features, such as a thinner fibrous cap and larger lipid-rich core. Women experience a rapid escalation of plaque instability and altered calcification pattern in the post-menopausal period, narrowing the gap in atherosclerotic risk between men and older women [22].

In a community-based prospective study of out-of-hospital sudden cardiac arrest (SCA) in the Pacific northwest in a population of middle-aged men and women (aged 35–65), men experienced a 7-fold increased incidence of sports-related SCA compared with women. The majority of SCA occurred during or within one hour of vigorous exercise including jogging, cycling, or basketball [23]. Histologic examination of sudden death victims (all with coronary artery thrombi, rupture, or severe coronary artery disease (CAD) without acute thrombus) revealed that women more frequently had plaque erosion and late-stage thrombi compared with men who more frequently had plaque rupture. Erosions tended to have smaller internal elastic lamina areas than ruptures and small or absent necrotic cores [24]. Evidence that the thrombotic healing process started before the symptomatic cardiac event offers potential insight into why women with coronary disease may present with more atypical symptoms and/or delayed presentations. While this pathophysiology primarily speaks to acute presentation, it underscores the importance of comprehensive risk factor modification through exercise, as erosion-prone plaque may be more responsive to the anti-inflammatory and metabolic interventions that exercise stimulates.

### Clinical Outcomes of Physical Activity on Coronary Artery Disease

The effects of exercise on women with CAD is understudied [19]. Women engaged in cardiac rehabilitation experience a reduction in chest pain and angina after myocardial infarction or coronary artery bypass grafting and reduction in repeat revascularization. Women with a history of spontaneous coronary artery dissection who engage in cardiac rehabilitation have an increase in exercise capacity without adverse events and a slight improvement in mental health scores [25]. In women with microvascular angina, eight weeks of cardiac rehab is associated with significant improvement in exercise tolerance, symptom severity, and psychosocial variables; gains which are maintained in follow up [26]. For women with ischemia with nonobstructive coronary artery disease, a pilot study of 62 women with BMI > 25 kg/m2 showed those randomized to weight loss, exercise and risk factor modification had significant improvement in anginal and depressive symptoms, weight loss, lipid profiles and A1c levels in diabetic compared to those who received standard of care [27]. Improvement in peak VO2 is inversely associated with mortality reduction and lower CAD events [28]. In a study of 83 patients with CAD (19 women, 64 men) engaged in different aerobic exercise training programs, Traschel et al. found men and women had a similar exercise training response measured by key CPET and echocardiographic parameters [29].

## Heart Failure

### Structural and Physiologic Changes

Myocardial structural changes occur in approximately 20–50% of patients with heart failure with reduced ejection fraction (HFrEF) in the form of sarcopenia and change in muscle fibers from type I to type IIb fibers. Decreased capillary density within each fiber, rapid depletion of high-energy phosphate and impairment in mitochondrial oxidative capacities occur in both HFrEF and heart failure with preserved ejection fraction (HFpEF) [30].

Lower exercise capacity is seen in women with heart failure compared with similar male patients across the range of ejection fractions. Evaluation of exercise parameters by means of echocardiographic and cardiopulmonary testing demonstrated worse LV compliance with higher filling pressures and decreased oxygen extraction in female patients with HFpEF [31]. Women have greater increase in pulmonary capillary wedge pressure relative to cardiac output, lower cardiac output reserve, more decreased pulmonary and systemic arterial compliance with exercise. Adverse pulmonary vascular remodeling and poorer diastolic reserve are associated with severity of symptoms in HFpEF [32]. With HFrEF and heart failure with mildly reduced EF (HFmrEF), smaller increases in LV end-diastolic volumes with exercise result in lesser increases in stroke volume and cardiac output in women leading to greater exercise intolerance [31].

Women are underrepresented in cardiovascular trials, including those in heart failure, limiting sex-specific analyses; consequently, evidence on sex-specific effects of exercise training remains limited. Nevertheless, the beneficial effects are recognized [33]. Reversal of LV remodeling, and modest improvement in LVEF and LV end-diastolic volume in heart failure patients has been associated with participation in endurance training however most studies enrolled males only or sex was not specified. Animal studies in pathological hypertrophy have shown upregulation of sarco/endoplasmic reticulum Ca2+-ATPase pump to improve contraction. Decrease in collagen deposition in male animals and decreased sympathetic activity and neuroendocrine hormones have also been shown with exercise however were not differentiated by sex [34]. Additionally, ET leads to a reduction in proinflammatory cytokines in skeletal muscle and increases the production of antioxidative molecules [35].

### Exercise Training and Heart Failure Effects

#### Heart Failure with Reduced Ejection Fraction

The effect of various modalities of exercise on reverse remodeling was assessed in the Study of Myocardial Recovery after Exercise Training in Heart Failure (SMARTEX-HF). Women comprised only 26% of the nonischemic group and 13% of the ischemic group. The impact of High Intensity Interval Training (HIIT) vs. Moderate Continuous Training (MCT) vs. regular exercise on cardiac parameters (left ventricular end-diastolic diameter and ejection fraction) and maximal exercise capacity (peak VO2) in HFrEF patients with ischemic and non-ischemic etiology were assessed. No significant effect on left ventricular size or LVEF or increase in peak VO2 was seen between etiologies or when assessed by exercise group and this persisted when age, sex and smoking status were included. There was a small but statistically significant within-group reduction in the primary endpoint of LVEDD at 12 weeks in the HIIT group, but not in the MCT group and did not differ by sex. The difference in peak VO2 between ICM and NICM was similar across sex (*p* = 0.67) [36].

In the landmark randomized trial HF-ACTION, (Heart Failure-A Controlled Trial Investigating Outcomes of Exercise TraiNing) only 28% of participants were women however sex-specific analysis was prespecified. Exercise training was compared with usual care in 2,331 patients with HFrEF and class II-IV symptoms. At baseline, women had 7% to 10% lower peak VO2 and 6-min walk distance than men. After analysis adjusting for known prognostic variables (HF cause, duration of cardiopulmonary exercise test, left ventricular ejection fraction, Beck Depression Inventory II score, and atrial fibrillation or flutter), exercise training was associated with a lower risk of all-cause mortality or hospitalization. In women there was no significant difference in peak VO2 with ET compared to men. However, women, who had more nonischemic etiology of HFrEF, had a greater reduction in the combined endpoint of all-cause mortality and hospitalization of 26% compared with men [37].

Assessing the addition of supervised center-based ET to a disease management program and home exercise program in patients recently discharged after heart failure hospitalization in a mixed population of HFrEF and HFpEF patients in the EJECTION-HF Randomized Phase 4 Trial with 25.5% female, there was no difference in the primary outcome of all-cause 12-month death or readmission. There was no difference in outcomes or treatment effect by sex but a trend toward greater benefit in participants age < 70 years. Guideline-based exercise was associated with a significantly lower rate of death and readmission. Notably, HFpEF patients had higher death and readmission overall, but there was no interaction with the allocation group [38].

#### Heart Failure with Preserved Ejection Fraction

As HFpEF, has a preponderance in older women, representation of women in some trials has been more significant. Many of these trials assessed exercise capacity, quality of life and echocardiographic parameters of elevated filling pressures. In a metanalysis of 8 studies of patients with HFpEF, exercise training improved VO2 max and quality of life measures but did not significantly change echocardiographic diastolic parameters (e’ and E/e’) or LVEF [39]. A more recent study, which was comprised of 63% women, comparing the effect of types of exercise training, moderate continuous training vs high intensity interval training on exercise capacity showed similarly, though non-significant, increases in VO2 max but did not show any difference between exercise modalities [40]. There was superiority of HIIT training over MCT in improving VO2 max (22% vs 11%) but both modalities were equal in improvement in quality of life, ventilatory efficiency and other CPET measures and diastolic function with decrease in E/e’ ratio [41]. Findings regarding improvement in E/e’ ratio, however, have been inconsistent. In a study combining endurance training and resistance training in HFpEF patients, the primary endpoint was a modified Packer score, including all-cause mortality, HF hospitalizations and changes in peak oxygen consumption (VO2), E/e’, New York Heart Association (NYHA) class and global self-assessment (GSA), 59.6% of participants were women. Improvement in peak VO2 and NYHA class were seen; however, there was no significant differences in E/e′, change in GSA, time to cardiovascular hospitalization or all-cause mortality [42].

### Quality of Life

Studies of determinants of Quality of life (QOL) in women with heart failure have also shown conflicting data with some showing association with exercise capacity. Others, including a pooled secondary analysis of RELAX and NEAT-HFpEF trials, showed association of QOL with 6-minute walk test (6MWT) only in men. QOL in women, as determined by Minnesota Living with Heart Failure Questionnaire, was associated only with age and BMI. Depression was not evaluated in this analysis and higher rate in women has been proposed as a factor in this discrepancy along with greater effect of social determinants of health in women [43].

### Recommendations for Exercise Training in Heart Failure

Based on trial data, there is a class I recommendation for ET or regular physical activity in HF patients in the 2022 American Heart Association/American College of Cardiology/Heart Failure Society of America guidelines [43]. In the 2024 American College of Cardiology Expert Consensus Decision Pathway for Treatment of HFrEF, there was further recommendation for aerobic ET [44]. For HFpEF, though ET has been associated with improved cardiorespiratory fitness and QOL, there are no definitive recommendations for ET in this group.

## Cardiac Rehabilitation

Cardiac rehabilitation (CR) offers significant benefits for women with cardiovascular disease, including lower mortality rate, fewer recurrent events and hospital readmissions, improved exercise capacity, reduced symptoms, and improved quality of life and psychosocial health [25]. It has been shown to reduce cardiovascular mortality by 26% with a dose-response association between the degree of adherence to the program and mortality. Exercise-based CR studies in coronary heart disease (CHD) have shown reductions in cardiovascular mortality, hospitalizations, and myocardial infarction, with favorable effects on QOL and cost-effectiveness. These findings that are relevant and of importance to women, who are traditionally underrepresented in most cardiovascular clinical trials, are poorly recognized, underdiagnosed, and under-treated [45]. Recently, the American Heart Association, in a scientific statement, reiterates CR as a Class I, guideline-supported intervention across indications relevant to women, and concludes that women who participate in CR achieve at least equivalent, and in some cohorts relatively greater, mortality risk reduction than men, with consistent improvements in blood pressure, lipids, glycemic control, tobacco cessation, and functional status, while highlighting persistent gaps in referral, enrollment, and completion [25].

Despite its benefits, cardiac rehabilitation is underutilized worldwide. It is known that on average, less than 10% of all participants in cardiac rehabilitation programs are women since they are less likely to be referred, and once they are referred, they are 36% less likely to participate than men [46, 47]. Barriers to cardiac rehabilitation are present in referral, enrollment, and adherence to the program [47, 48]. According to the results of the first global survey conducted on barriers to cardiac rehabilitation by sex, womens’ greatest obstacles to enrollment included lack of awareness, cost, and difficulty exercising due to pain or perceived tiredness. Other impediments to adherence included distance, transportation, and family responsibilities. Women who are not employed encounter distinct challenges in accessing cardiac rehabilitation [48]. Previous research has also highlighted barriers including limited educational attainment, social isolation, absence of insurance coverage, as well as mental health, medical, psychosocial, and behavioral factors [49].

Sex adaptation of cardiac rehabilitation involves adapting content, mode and/or composition by sex, which improves women’s adherence and mental health outcomes compared to traditional programs. However, according to the results of the global study on programs with adaptation for women, sex-adapted programs are not commonly offered. In 93 countries with cardiac rehabilitation, only 11.8% of programs offered women-only cardiac rehabilitation programs, and these were the largest and well-resourced programs [50, 51].

## Conclusions

Women derive greater cardiovascular and mortality benefits from physical activity than men, even with lower exercise volumes. Biological and hormonal differences contribute to these outcomes, but women face significant barriers to cardiac rehabilitation and are underrepresented in research. Tailored exercise recommendations and improved access to rehabilitation are essential to maximize cardiovascular health for women.

## Key References

- Ji H, Gulati M, Huang TY, Kwan AC, Ouyang D, Ebinger JE, et al. SexDifferences in Association of Physical Activity With All-Cause andCardiovascular Mortality. J Am Coll Cardiol. 2024;83(8):783-93.
  - ○ Findings of this study suggest that women derive greater benefitfrom an equivalent amount of exercise compared to men withrespect to cardiovascular and all-cause mortality.
- Ghisi GLM, Kim WS, Cha S, Aljehani R, Cruz MMA, Vanderlei LCM, et al.Women's Cardiac Rehabilitation Barriers: Results of the International Councilof Cardiovascular Prevention and Rehabilitation's First Global Assessment.Can J Cardiol. 2023;39(11s):S375-s83.
  - ○ Findings of this study, show variability in barriers to cardiacrehabilitation vary by geographical location and were greater inunemployed vs employed women.

## Data Availability

No datasets were generated or analysed during the current study.
